# Assessment of prevalence, knowledge and health-related practices of dysmenorrhea among Malaysian women in Kuala Lumpur: a cross-sectional survey

**DOI:** 10.1080/07853890.2023.2281655

**Published:** 2023-11-27

**Authors:** Rana Mohamad Bakro, Muhammad Junaid Farrukh, Mogana Rajagopal, Susi Ari Kristina, Diana Laila Ramatillah, Long Chiau Ming, Ganesh Sritheran Paneerselvam, Muhammad Abdul Hadi

**Affiliations:** a Faculty of Pharmaceutical Sciences, UCSI University, Kuala Lumpur, Malaysia; b Department of Pharmaceutics, Faculty of Pharmacy, Universitas Gadjah Mada, Yogyakarta, Indonesia; c Faculty of Pharmacy, Universitas 17 Agustus 1945, Jakarta, Indonesia; d School of Medical and Life Sciences, Sunway University, Sunway City, Malaysia; e Department of Hospital and Clinical Pharmacy, School of Pharmacy, Taylor’s University, Selangor, Malaysia; f College of Pharmacy, QU Health, Qatar University, Doha, Qatar

**Keywords:** Dysmenorrhea, knowledge, practices, Malaysia, socio-demographic

## Abstract

**Background:**

Menstruation is a natural phenomenon considered an important indicator of women’s health, reflecting their endocrine function. Women in low middle income countries face substantial menstrual hygiene management challenges. Data on the knowledge of dysmenorrhea and health-related practices among Malaysian women are scarce. The present study aimed to investigate the prevalence of dysmenorrhea among Malaysian women in Kuala Lumpur and its association with socio-demographic factors, knowledge level, and general practices.

**Method:**

A cross-sectional study was carried out among Malaysian women in Kuala Lumpur. A total of 362 unmarried women, nulliparous and aged between 18 and 25 years old, were included in this study. Participants were conveniently recruited through online platforms as well as face to face using a self-administered questionnaire with five sections consisting of demographics, menstrual characteristics, Working ability, Location, Intensity, Days of pain, Dysmenorrhea (WaLIDD) score for diagnosing and assessing the severity of dysmenorrhea as well as an evaluation of respondents’ general knowledge and practices towards dysmenorrhea. The collected data were analysed using the SPSS tool, a descriptive statistic was used to report demographic characteristics. Inferential statistics was used to report the differentiation, association, and correlations of the variables.

**Results:**

The prevalence of primary dysmenorrhea was 73.2%. It was found that the majority of the respondents had poor knowledge (60%) and poor practices (61.88%) of dysmenorrhea. The most common preventive practices among the respondents were using dietary supplements, and herbs, taking a rest and exercising. The findings also indicated that dysmenorrhea among the respondents was significantly associated with family history of dysmenorrhea (*p* = 0.002), monthly income (*p* = 0.001), and knowledge level (*p* = 0.001).

**Conclusion:**

Dysmenorrhea has a high prevalence among women in Malaysia in Kula Lumpur driven by low knowledge and lack of evidence-based practices among these women. Thus, it is critical for Government and healthcare authorities to promote education related to women health among Malaysian women.

## Introduction

Dysmenorrhea is defined as the presence of painful cramps of uterine origin that occur during menstruation and represents one of the most common causes of pelvic pain and menstrual disorder. The International Association for the Study of Pain defines pain as “an unpleasant sensory and emotional experience associated with actual or potential tissue damage, or described in terms of such damage [1,2]. As it is a period event that punctuates most women’s lives, menstruation, and its related issues should be taken into consideration during the discussion of women’s health promotion [3]. Several factors have been reported as risk factors for primary dysmenorrhea, such as family history, heredity, lower family income, weight, high stress, age of menarche, and smoking [4–7].

The prevalence of dysmenorrhea in the same population can significantly vary based on the selected criteria used for the assessment [8]. In Malaysia, primary dysmenorrhea prevalence reached a significant rate of 62.3% [9]. Although primary dysmenorrhea is not a real threat to life, it is one of the important health issues that must be addressed by health professionals as it can impact women’s quality of life [10]. The burden of primary dysmenorrhea is more significant than any other gynaecological problem. It is the primary source of gynaecological morbidity in women of reproductive age [11]. In Malaysia, dysmenorrhea plays a role in reducing concentration and increasing absenteeism from school or work. It has a negative impact on women’s activities and reproductivity [9] and in severe cases may lead to disability, inefficiency, or secondary psychological problems in some women, resulting in depression and leading to inactive engagement in multiple social relationships [12]. Moreover, the economic losses due to health care costs and decreased productivity.

The problem is well raised from an inadequate level of knowledge on dysmenorrhea, women with poor knowledge may follow poor health-related practices that led to improper management of dysmenorrhea and lack of menstrual hygiene practices, which may affect their quality of life [13]. poor practices can pose serious health risks like reproductive and urinary tract infections which can result in future infertility, studies confirm a clear and reliable correlation between inadequate menstrual hygiene practices and a greater prevalence of lower reproductive tract infections (RTIs) [14]. Also, poor management can include poor medication practices which increase the risk of comorbidities such as renal impartments or gastrointestinal bleeding that in acute cases though potentially life-threatening, as a result of pain killer abuse that is used to reduce abdominal cramps and pain in dysmenorrhea [15].

The prevalence of dysmenorrhea is high but women don’t have adequate knowledge about this. Improper management can lead to poor quality of life which can affect their academic and non-academic performance [8,10]. Most of the studies done in Malaysia emphasized on the prevalence of dysmenorrhea among Malaysian women but so far there is no study conducted in Malaysia to assess the knowledge and health-related practices of dysmenorrhea among Malaysian women. This study aimed to determine and interpret the prevalence of symptoms of dysmenorrhea, its knowledge and their health-related practices.

## Material and methods

### Study design and setting

A descriptive cross-sectional study was carried out among Malaysian women in Kuala Lumpur, Malaysia. Data was collected in the period from 1 January to 1 September 2021.

### Sampling method and sampling size

Multiple methods were utilized to approach target participants. The online survey was carried out on several social media platforms such as Facebook, Twitter, Snapchat, and Instagram using a link to google forms. At the same time, the questionnaire had been physically distributed in the Kuala Lumpur region, at public area metro stations, restaurants, food trucks, salons, shops, and malls.

The total population of women in the Federal Territory of Kuala Lumpur in 2018 according to the Department of Statistics Malaysia was approximately 870,000 [16]. The sample size was determined using the Raosoft sample size calculator [17]. A confidence level of 95%, a margin of error of 5%, and a response distribution of 50% were selected. Based on this calculation, the sample size needed is 384. After factoring in a 20% non-response rate, the required sample size becomes 461.

## Inclusion criteria

Malaysian nulliparous unmarried women aged between 18 and 25 years were included in this study.

## Exclusion criteria

Foreigners and married women were excluded.

### Study tool

The instrument used in this study was a self-administered questionnaire. The questionnaire was adapted from other published studies [3,18]. The questionnaire consisted of five sections. Section A consisted of ‘participants’ demographic characteristics. Section B was screening questions for primary dysmenorrhea including the WaLIDD instrument. It’s a scale designed by Teherán et al. that’s used to diagnose and assess the severity of dysmenorrhea. It is a scale-type instrument that includes (working ability, location, intensity, and days of pain) which integrated features of dysmenorrhea such as 1) several anatomical pain locations (no part of the body, lower abdomen, lumbar region, lower limbs, inguinal region), 2) a Wong-Baker range (a numeric pain scale was reclassified to adjust a four-level does not hurt, hurts a little, hurts a little more, hurts, even more, hurts a lot, hurts a lot more), 3) a number of days of pain during menstruation (0, 1–2, 3–4, ≥5), and 4) frequency of restricting pain to perform their activities (never, almost never, almost always, always) [18, 19]. Section C was to determine menstrual characteristics, Section D was to assess the knowledge scale among the respondents, and Section E was to assess their general practices to-wards dysmenorrhea.

### Scoring criteria

There were a total of 12 knowledge items, each item had 3 choices, including: True, False, and Not sure. The scoring method was used for analysis. The correct answer was given a score of 3, not sure was given a score of 2, while the incorrect answer was scored at 1, with a maximum total score of 36. A knowledge score below the mean was classified as poor knowledge, and a score of more than the mean was classified as good knowledge [20]. For general practices, 14 items were included based on a Likert scale rating of five points. The Likert scale for practices includes never (score 1), rarely (score 2), sometimes (score 3), frequently (score 4), and always (score 5). The possible score for practices ranged from 1 to 70, where respondents with a score below the mean were reported as having poor practices, and those with a score above the mean were recorded as having good practices [20]. To assess the severity of dysmenorrhea, a numeric pain scale was reclassified to adjust to a four-level.

### Validation and pilot study

The questionnaire was sent to five experts (physicians, academicians, and pharmacists) for Content validity. The questionnaire was revised based on reviewer feedback until it was ready for the pilot study. After completing the face validity process, a pilot study was carried out on 30 participants to ensure the reliability of the questionnaire formulated. The internal consistency was calculated by Cronbach’s alpha coefficient, where it was found that Cronbach’s alpha score for both knowledge and general practice items was within the acceptable range, recording a score of 0.61 and 0.70, respectively. Two questions had been excluded from the questionnaire, so the final draft was consisting of −57- valid questions.

### Statistical analysis

Statistical analysis was performed using IBM SPSS Statistics Version 20. The obtained data were analyzed using descriptive and inferential analysis, and a normality test was also performed prior to data analysis. The data obtained were analyzed using descriptive and inferential analysis where percentage, mean and standard deviation were used to report demographic characteristics, knowledge, and practice scores. Whereas *P*-value and Chi-square tests were used to analyze the differentiation, association, and correlations of the study variables. In the final model, only variables with *p* < 0.05 were considered to have a significant influence on dysmenorrhea prevalence. The significant variables with a *p*-value <0.05 in the univariate analysis were selected to be modeled using a simple logistic regression method for the estimation of Odds ratios (OR).

## Results

Out of 400 respondents who were approached, about 362 respondents participated in the study, which gives it a response rate of 90.5%. Table 1 shows that out of 362 respondents, most of the respondents were Malays (around 50%), and the mean (SD) age of respondents was 22.33 (1.91) years. The results also showed that most of the respondents (65.2%) were university students. It was also found that 45.3% of the respondents had a family history of dysmenorrhea. The detailed demographic description of the respondents is represented in Table 1.

**Table 1.** Socio-demographic characteristics among the respondents (*n* = 362).

Socio-demographic	Frequency (*n*)	Percentage (%)
Age group (years)		
18–22	188	51.9
23–25	174	48.1
Ethnicity		
Malay	186	51.4
Chinese	157	43.4
Indian	19	5.2
Level of education		
Primary school	5	1.4
Secondary school	44	12.2
College	75	20.7
University	236	65.2
Other	2	6
Total Family Monthly Income (RM)		
≤999	233	64.4
1000–2999	67	18.5
≥3000	28	7.7
No income	34	9.4
Employment status		
Employee	146	40.3
Unemployed	216	59.7
Family history of dysmenorrhea		
With family history	164	45.3
Without family history	198	54.7

In this study, one hundred eighty-eight (51.9%) respondents reported having regular menstruation. As shown in Table 2, among the 362 respondents, around (51.4%) showed a normal menstrual cycle duration of between 21 to 35 days, while (43.4%) of the respondents had a cycle length of fewer than 21 days and only 5.2% of them had a menstrual cycle longer than 35 days. A moderate menstrual flow was observed among (64.1%) of the respondents, whereas a light menstrual flow was recorded in (30.1%) of them, and very few respondents (5.8%) had a heavy menstrual flow. For the primary dysmenorrhea accompanying symptoms, the results indicated that low back pain, abdominal cramps, and breast discomfort were the most frequently recorded symptoms in 65.2%, 57.2%, and 42% of the respondents, respectively.

**Table 2.** Analysis of menstrual characteristics of the respondents.

Menstrual characteristics	Frequency (*n*)	Percentage (%)
Regularity		
Regular	188	51.9
Irregular	174	48.1
Length		
≤21 days	157	43.4
21–35	186	51.4
≥35 days	19	5.2
Duration		
< 5 days	54 (14.9)	14.9
5–7 days	276 (76.2)	76.2
≥8	32 (8.8)	8.8
Menstrual flow		
Light	109	30.1
Moderate	232	64.1
Heavy	21	5.8
Accompanying symptoms		
Abdominal cramps	209	57.7
Low back pain	236	65.2
Pain in the upper thighs	66	18.2
Headache or migraines	132	36.5
Aches all over	57	15.5
Bloating	129	35.6
Breast discomfort	152	42.0
Nausea and vomiting	56	15.5
Reduced appetite	93	25.7
Diarrhea	84	23.2
Constipation	40	11
Hot flashes	23	8.8

According to Table 3, this study recorded a high prevalence of dysmenorrhea among the respondents (71.8%) in the age group 18 to 22, where the majority of them (53.2%) had moderate severity. In addition, the results showed that dysmenorrhea is much more prevalent among the Indian ethnicity, where 78.9% of the Indian respondents had dysmenorrhea compared to a prevalence of 76.3% and 68.8% observed in the Malay and Chinese respondents, respectively. Moreover, it was found that dysmenorrhea is more severe among Indian women (47.4%). Overall, most of the respondents (51.38%; *n* = 186) had moderate dysmenorrhea, whereas 25.97% (*n* = 94) had severe dysmenorrhea, and 22.65% (*n* = 82) of the respondents had mild dysmenorrhea.

**Table 3.** Prevalence and severity of dysmenorrhea by age and ethnicity.

	Dysmenorrhea		Severity		
Variable	Yes	No	Mild	Moderate	Severe
Age, *n* (%)					
18 − 22	135 (71.8)	53 (28.2)	46 (24.5)	100 (53.2)	42 (22.3)
23–25	130 (74.7)	44 (25.3)	36 (20.7)	86 (49.4)	52 (29.9)
Race, *n (%)*					
Malay	142 (76.3)	44 (23.7)	34 (18.3)	92 (49.5)	60 (32.3)
Chinese	108 (68.8)	49 (31.2)	46 (29.3)	86 (54.8)	25 (15.9)
Indian	15 (78.9)	4 (21.1)	2 (10.5)	8 (42.1)	9 (47.4)

As shown in Table 4, the majority of respondents (68%) did not have awareness of dysmenorrhea care, where it was found that only 32% had awareness of dysmenorrhea care. The findings presented in Table 4 also showed that 29.8% (*n* = 108) of the respondents got their first care from a family doctor and 29.3% (*n* = 106) of them got their first care from a pharmacist. The other respondents got their first care from a therapist (19.3%), a gynecology specialist (12.4%), or a school nurse (9.2%).

**Table 4.** ‘Patient’s awareness and dysmenorrhea history.

‘Patients’ awareness and dysmenorrhea	Frequency (*n*)	Percentage (%)
Awareness		
Yes	116	32
No	246	68
First dysmenorrhea care		
Family doctor	108	29.8
Gynecology specialist	45	12.4
School nurse	33	9.2
Therapist	70	19.3
Pharmacist	106	29.3

Overall, the majority of the respondents (60%, *n* = 217) had low knowledge of dysmenorrhea. More than two-thirds of the respondents did not have accurate information about the exact age of the menstrual cycle, and only one-third knew about the menstrual period. Moreover, only 21.3% of them knew that the ovum that was released during ovulation comes out together with the menstrual blood. The findings of different items used to assess the knowledge of dysmenorrhea among the respondents are presented in Table 5.

**Table 5.** Items analysis of knowledge on dysmenorrhea.

Knowledge on dysmenorrhea	True *n* (%)	False *n* (%)	Not sure *n* (%)
Most girls start their periods when they are about 12 years old.	123 (34)	132 (36.8)	107 (29.2)
Menstrual blood comes from the inner lining of the uterus through the vagina	125 (34.6)	137 (38.4)	100 (27)
The normal days for menstrual flow usually occur every 21 to 35 days, and blood flow lasts two to seven days.	122 (34.3)	174 (41.6)	91 (24.1)
The ovum that was released during ovulation comes out together with the menstrual blood	83 (21.3)	162 (46.3)	117 (32.4)
At an average age of 45 - 58 women stop menstruating	118 (32.4)	153 (43.5)	91 (24.1)
Dysmenorrhea symptoms include [low back pain, abdominal pain nausea, vomiting, change in bowel movements, change in appetite]	133 (37.1)	135 (37.8)	94 (25.1)
Changes in a ‘woman’s routine such as long working hours or the lockdown during covid 19, can cause changes in the menstrual cycle	134 (40.6)	138 (38.7)	94 (25.1)
Some ‘woman experience mood swings during menarche	173 (49.8)	121 (33.3)	68 (16.8)
Taking painkillers such as "mefenamic acid" frequently can cause kidney damage and stomach bleeding.	114 (31.1)	163 (46.7)	95 (22.2)
Maximum paracetamol tablets can be taken up to 8 tablets per day, of 500 mg paracetamol	124 (34.3)	147 (41.6)	91 (24.1)
Long-term consumption of oral contraceptives can reduce periods of pain, but increase the risk of cervical cancer	83 (21.3)	162 (46.3)	117 (32.4)
Long-term consumption of pain killers can cause infertility	118 (32.4)	153 (43.5)	91 (24.1)

More than half (61.88%) of the respondents had poor general practices and only 38.12% of them had good practices towards dysmenorrhea. The results indicated that some practices were common among the respondents, such as using dietary supplements (54.6% frequently, 5.7% always), using herbs (50.2 frequently, 5.1% always), taking a rest (45.7% frequently, 9.5% always), and reducing salt (44.4% frequently, 14.6% always). On the other hand, some practices such as using relaxation methods and following a special diet were less frequent among the respondents. Where 38.4% of respondents stated that they rarely use relaxation methods and 1.3% of them have never used them. In addition, 50.8% of the respondents stated that they rarely use a special diet, and 13.7% of them have never used a special diet as a practice for dysmenorrhea management. Exercising was also one of the most frequent practices among the respondents towards dysmenorrhea; where 35.9 of them reported that they do exercise frequently and 11.4% of them do it always. Changing the pad at least every 3 or 4 h as an important hygiene practice towards dysmenorrhea was recorded frequently or always among 35% of the respondents. The analysis of the different items for measuring the practices toward dysmenorrhea is presented in Table 6.

**Table 6.** Items analysis of practices towards dysmenorrhea.

Variables	Never	Rarely	Sometimes	Frequently	Always
	*n (%)*	*n (%)*	*n (%)*	*n (%)*	*n (%)*
I use dietary supplements (e.g. vitamins\minerals, fish oil	15 (1.9)	36 (8.3)	103 (29.5)	181 (54.6)	27 (5.7)
I use herbs (e.g. ginger, cinnamon, Chinese traditional herpes)	12 (0.6)	45 (11.1)	113 (33)	166 (50.2)	25 (5.1)
I use relaxation methods (e.g. deep breathing, progressive relaxation, meditation	14 (1.3)	131 (38.4)	126 (37.1)	47 (12.1)	44 (11.7)
I try acupressure or seeking massage	0 (0.0)	58 (12.7)	126 (37.1)	131 (38.4)	74 (11.7)
I do exercises	12 (1.6)	63 (17.1)	117 (34)	123 (35.9)	45 (11.4)
I take a rest (e.g. lying down, sleeping)	13 (1.3)	29 (6.3)	105 (30.2)	153 (45.7)	40 (9.5)
Distraction (e.g. listening to music, watching TV…)	14 (1.6)	34 (7.6)	184 (55.6)	96 (27.3)	34 (7.9)
I follow a special diet	52 (13.7)	169 (50.8)	84 (23.5)	47 (12.1)	48 (12.1)
I reduce my salt intake.	15 (1.9)	35 (8.3)	106 (30.8)	150 (44.4)	56 (14.6)
I reduce caffeine, consumption.	11 (0.6)	38 (9.2)	173 (52.1)	106 (30.5)	34 (7.6)
I use heat (e.g. heating pads, hot shower\ bath)	0 (0.0)	48 (9.5)	116 (34)	165 (49.2)	33 (7.3)
I use a TENS machinal	13 (1.3)	47 (12.1)	106 (30.8)	150 (44.4)	46 (11.4)
I Change my pad at least every 3 or 4 hours.	14 (1.6)	63 (17.1)	116 (34)	123 (35.9)	46 (14.4)
I wear comfortable, loose clothing, rather than jeans or tight-fitting during periods.	14 (1.6)	52 (13.7)	101 (29.5)	154 (45.7)	40 (9.5)

As shown in Table 7, the chi-square analysis result showed that there was no significant association between dysmenorrhea and age group (χ2 = 0.388, *p* = 0.553), and between education and dysmenorrhea (χ2 = 0.091, *p* = 0.767). The same observation was recorded for employment status, where an insignificant relationship was noted between dysmenorrhea and employment status (χ2 = 0.001, *p* = 0.976). Meanwhile, a significant relationship was observed between dysmenorrhea prevalence and monthly income (χ2 = 21.81, *p* = 0.001), and between dysmenorrhea prevalence and family history (χ2 = 9.52, *p* = 0.002).

**Table 7.** Association between dysmenorrhea and socio-demographic factors.

	Dysmenorrhea status			
Variable	Yes (%)	No (%)	χ 2	*P*
Age group				
18–22	135 (71.8)	53 (28.2)	.388	.533
23–25	130 (74.7)	44 (25.3)		
Education				
≤low level	35 (71.4)	14 (28.6)	.091	.762
≥high level	230 (73.5)	83 (25.5)		
Employment status				
Unemployed	158 (73.3)	58 (25.7)	0.001	.976
Employed	107 (73.3)	39 (72.6)		
Monthly income				
≤1499	218 (78.7)	59 (21.3)	18.16	.001*
≥1500	47 (55.3)	38 (44.7)		
Family history				
With FH	133 (81.1)	31 (18.9)	9.52	.002*
Without FH	132 (66.7)	66 (33.3)		

Note: χ ^2^= chi-square value. Significance level *p* < 0.05.

There was a significant association between dysmenorrhea and knowledge and practice levels (*p*=.001*, .007*) respectively, where the majority of the respondents with dysmenorrhea had low knowledge and poor practices, as shown in Table 8.

**Table 8.** Association between dysmenorrhea and knowledge and general practices.

	Dysmenorrhea		χ2	*P*
Variables	Yes (%)	No (%)		
General knowledge				
Poor	174 (80.2)	43 (19.8)	13.455	.001*
Good	91 (62.8)	54 (37.2)		
General practices				
Poor	175 (78.1)	49 (21.8)	7.253	.007*
Good	90 (20.8)	48 (65.2)		

Note: χ ^2^= chi-square value. Significance level *p* < 0.05.

Simple logistic regression analysis results in Table 9 showed that those having low income are 42.3% times higher odds of having dysmenorrhea (OR = 1.423), the findings also indicated that respondents with a family history of dysmenorrhea are 21.6% more likely to be exposed to dysmenorrhea. It was also found that the prevalence of dysmenorrhea is 0.423 times (OR = 1.423) higher among the respondents with low monthly income compared to those with high monthly income. Generally, respondents with low general knowledge are 0.28 times more likely (OR = 1.28) to have primary dysmenorrhea, and having poor general practice increases the odds of having primary dysmenorrhea by 0.2 times (OR = 1.2).

**Table 9.** Results of the simple logistic regression crude or.

Predictors	Crude OR	95% CI	*P* value
Family history (With FH)	1.216	.885–1.671	.002*
Low monthly income	1.423	.956–2.119	.001*
General knowledge (poor)	1.280	.919–1.776	.001*
General practices (poor)	1.200	.860–1.668	.007*

Significance level *p* < 0.05. OR: odd ratio.

## Discussion

The current study was conducted to determine the socio-demographics of Kuala Lumpur respondents. The prevalence of dysmenorrhea among respondents and the influence of both knowledge and practice. About 362 respondents participated in this study, the age ranging from 18 years to 25 means (SD) 22.33(1.91), and the majority (51.4%) were Malay (details are in Table 1). The prevalence of dysmenorrhea is difficult to determine because of the different definitions of the condition. The estimates vary from 45% to 95% [21]. This study showed that the majority of respondents (73.2%) have primary dysmenorrhea and about 26% of them have severe dysmenorrhea. Previous studies conducted in India have reported a similar prevalence rate of 84.2%. For instance, the study in Italy was about 81.4% prevalence [22]. This high prevalence rate is related to multiple factors such as lifestyle, age, and socio-economic status as well as the effectiveness of the control and preventive measures put in place to the severity of dysmenorrhea, especially among the younger age group [23].

The current study shows that moderate dysmenorrhea among the age group 18–22 years old, had the highest prevalence at 53.2% compared to 49.2% among the age group 23–25 years old, followed by mild dysmenorrhea at 24.5% among the age group 18–22 years old, and severe dysmenorrhea, which was recorded among 22.3% of the respondents in the age group 18–22. These findings are in line with previous studies which reported age as one of the major factors that influence the severity of dysmenorrhea [24]. This difference in the prevalence of severe dysmenorrhea in Malaysia can be understood due to the multicultural population and medication practice diversity. The current study demonstrated that about 45.3% of the respondents reported that they have a family history of dysmenorrhea; this rate is close to the findings of the study conducted by Abubakar et al. which reported that 50.7% of Malaysian women have a family history of dysmenorrhea [25].

The study of clinical menstrual characteristics among the respondents showed that the majority of them had a regular menstrual cycle (51.9%), for a duration of between 5 and 7 days (76.2%) with a length of between 28 and 35 days (51.4), which is characterized by moderate menstrual flow (64.1%). These findings are in agreement with the study conducted by Lee et al.to, investigate the menstrual irregularities among Malaysian girls, which reported that 62.8% of the respondents had a regular menstrual cycle [26]. On the other hand, 65.2% of the respondents experienced low back pain, 57.2% of them had abdominal cramps, and 42% of them experienced breast discomfort as accompanying symptoms. In the same context, a previous study conducted to investigate the prevalence of dysmenorrhea and its sequel among medical students at a Malaysian University showed that low back pain (53.7%), abdominal cramps (47.6%), and breast discomfort (40.9) are the most frequent symptoms among the respondents [27].

The current study showed that there was no association between dysmenorrhea and some socio-demographic variables elements such as age group, education, and employment status. However, there was a significant relationship between dysmenorrhea and other socio-demographic variables such as monthly income and family history. A similar result was reported with no association between elements of sociodemographics and dysmenorrhea. In Ethiopia, Gebeyehu, et al. reported no association between education and dysmenorrhea *p* = 0.210 [28]. In Turkey reported occupation and education as not associated with dysmenorrhea χ 2 = 1.294, *p* = 0.255 and χ 2 = 1.294, *P* = 0.255 [24]. On the contrary, certain studies have reported an association between elements of socio-demographics and dysmenorrhea. For instance, In Ethiopia, Tadese, et al. reported that occupation was significantly associated with dysmenorrhea *p* < 0.001 [29]. In another study conducted by Charu et al. in India, it was reported that there was a significant association between dysmenorrhea and socio-demographic variable elements (age group, family monthly income, and family history) [4]. A similar result was reported in some of these elements of socio-demographic factors. In Ita-ly, Grandi et al. reported age group, education, and family monthly income [22]. Latte et al. conducted a study in the USA and reported the impact of age group on dysmenorrhea prevalence [30]. The variation between those studies and this study could be due to the variable and operational definitions employed. For example, poor education in our study refers to an education level less than a university, whereas in other studies, Ethiopia and Turkey have different definitions. This also applies to the categorization of age and family income.

As shown in this study, only 40% of respondents had good general knowledge. In a similar report in Sri Lanka, only 14% of respondents had good general knowledge [31]. On the contrary, Mouhammad & Farzaneh, reported that 69.5% of the women in Iran had good general knowledge [32]. Meanwhile, 58% of respondents in Turkey had good knowledge of dysmenorrhea [33]. Another study conducted by Asyikin et al. showed that 73.2% of women adults in the north-eastern state of Peninsular Malaysia had a good knowledge of dysmenorrhea; this high proportion was recorded due to the inclusion of some Islamic ruling items in the questionnaire while the majority of the population had an Islamic background [34].

In the present study, knowledge of dysmenorrhea was significantly associated with dysmenorrhea prevalence (χ2 = 13.455, *p* = 0.001). The same findings were reported by Ghanaie et al. indicating the significant relationship between knowledge and dysmenorrhea in Iranian women; they have also reported that the knowledge level of patients on primary dysmenorrhea could be affected by their level of education [35].

Concerning general practice toward dysmenorrhea, this study shows that the majority of respondents (61.88%) had poor practices toward dysmenorrhea. As seen in this study, primary dysmenorrhea was statistically associated with general practice (χ2 = 7.253, *p* = 0.007). In contrast, Ghanaie et al. reported that there were no significant differences in practice scores in terms of dysmenorrhea, this is due to the fact the respondents were medical sciences students that had the same education level [35]. It was previously reported that poor practices among patients are related to poor sociodemographic and environmental factors, For example, a low education level is reflected in low knowledge; in turn, it will lead to poor practices toward dysmenorrhea [31].

The simple logistic regression analysis indicated that respondents with a family history of dysmenorrhea were 1.2 more likely to have primary dysmenorrhea (OR = 1.216, 95% CI = 0.885–1.671, *p* = 0.002). These results are in agreement with the findings reported in previous studies [36,37]. The association between dysmenorrhea and family history has been explained in the literature using two approaches. The genetic theory claims that the severity of dysmenorrhea is related to chromosome 1p13.2 located near the locus of nerve growth factor (NGF) which plays a key role in the inflammatory reflex and increases the irritability of the body [38]. On the other hand, the behavioral theory explains this association by the similarity of behavior and learning processes among the same family members, where the mother is considered the main source of knowledge and practices of the child; this similarity makes the mother and child usually experience the same severity of menstrual pain [39].

The current study also showed that monthly income levels had a significant influence on the prevalence rate of primary dysmenorrhea, respondents with low monthly income were 1.42 times more likely to have primary dysmenorrhea compared to those with high monthly income (OR = 1.423, 95% CI = 0.956–2.119, *p* = 0.001). This result could be due to having lower access to health services for this group compared to those having a higher monthly income. A previous study conducted among women students in a Turkish university indicated students with satisfactory stipend allowance had ∼1.5 times higher prevalence of dysmenorrhea [40] In contrast, another study conducted among Ethiopian students indicated that the prevalence of dysmenorrhea was 7 times higher in the respondents with low monthly income compared to those with high monthly income [41].

It was also observed that respondents with low general knowledge of dysmenorrhea were 1.28 times more likely to have primary dysmenorrhea (OR = 1.28, 95% CI = 0.919–1.776, *p* = 0.001). This could be attributed to the relationship between good knowledge regarding dysmenorrhea and its good management [27]. On the other hand, Ghanaie et al. reported the association between good knowledge and the prevalence of dysmenorrhea, and they explained the interest of people with dysmenorrhea in obtaining information in this field [35].The findings of this study also indicated a significant association between general practices and primary dysmenorrhea where the prevalence of dysmenorrhea in respondents with poor practices was 1.2 higher compared to those having good practices (OR = 1.2, 95%CI = 0.860–1.776, *p* = 0.007). Similar results were reported in Sri Lanka, where simple logistic regression showed that respondents with poor general practice toward dysmenorrhea were 3 times more likely to have severe dysmenorrhea (OR = 2.783, 95% CI = 1.627–4.760, *p* = 0.001) [31]. Thus, it is recommended to enhance counseling education to help women to cope with dysmenorrhea [35].

Good menstrual hygiene management (MHM) plays a fundamental role in enabling women, girls, and other menstruators to reach their full potential. In Malaysia, there is allocation of RM10 million in Budget 2022 by the Malaysian Government to provide a free monthly supply of pads to 130,000 girls from low socioeconomic status, in parallel with increased education and awareness on menstrual hygiene and management [42]. Also, menstrual products were previously taxed 6%. Malaysia Finally Lifts tax on Menstrual Hygiene Products [43].

## Limitations

This research was conducted online *via* multiple social media platforms, there is a possibility of biasness toward some underprivileged populations (aborigines/orang asli) that may not have access to social media Another limitation found in this study is that the respondents may answer questions based on what they think is right instead of what they have been practicing in their daily lives. Data was collected by convenient sampling, which is non-probabilistic nature of the sampling strategy, which is vulnerable to selection bias.

## Conclusion

Dysmenorrhea was significantly associated with socio-economic factors such as family history and monthly income, poor practice, and low knowledge. Enhancing health education for adolescent girls and implementing routine screenings by healthcare pro­viders can effectively limit dysmenorrhea’s negative impact. Future research should explore parental education and psychological factors’ influence on women’s attitudes toward dysmenorrhea management, aiding in the development of strategies to promote healthcare-seeking over self-medication.

## Data Availability

The datasets generated during and/or analyzed during the current study are available from the corresponding author on reasonable request.
